# Metabolites of neuroinflammation relate to neuropathic pain after spinal cord injury

**DOI:** 10.1212/WNL.0000000000010003

**Published:** 2020-08-18

**Authors:** Dario Pfyffer, Patrik O. Wyss, Eveline Huber, Armin Curt, Anke Henning, Patrick Freund

**Affiliations:** From the Spinal Cord Injury Center (D.P., E.H., A.C., P.F.), Balgrist University Hospital, University of Zurich; Institute for Biomedical Engineering (A.H., P.O.W.), University and ETH, Zurich; Department of Radiology (P.O.W.), Swiss Paraplegic Centre, Nottwil, Switzerland; Max Planck Institute for Biological Cybernetics (P.O.W., A.H.), Tuebingen, Germany; Advanced Imaging Research Center (A.H.), UT Southwestern Medical Center, Dallas TX; Department of Brain Repair and Rehabilitation (P.F.) and Wellcome Trust Centre for Neuroimaging (P.F.), UCL Institute of Neurology, University College London, UK; and Department of Neurophysics (P.F.), Max Planck Institute for Human Cognitive and Brain Sciences, Leipzig, Germany.

## Abstract

**Objective:**

To determine whether cervical cord levels of metabolites are associated with pain sensation after spinal cord injury (SCI) by performing magnetic resonance spectroscopy in patients with SCI with and without neuropathic pain (NP).

**Methods:**

Cervical cord single-voxel spectroscopic data of 24 patients with SCI (14 with NP, 10 pain-free) and 21 healthy controls were acquired at C2/3 to investigate metabolite ratios associated with neuroinflammation (choline-containing compounds to myoinositol [tCho/mI]) and neurodegeneration (total N-acetylaspartate to myo-inositol [tNAA/mI]). NP levels were measured, and Spearman correlation tests assessed associations between metabolite levels, cord atrophy, and pinprick score.

**Results:**

In patients with NP, tCho/mI levels were increased (*p* = 0.024) compared to pain-free patients and negatively related to cord atrophy (*p* = 0.006, *r* = 0.714). Better pinprick score was associated with higher tCho/mI levels (*p* = 0.032, *r* = 0.574). In pain-free patients, tCho/mI levels were not related to cord atrophy (*p* = 0.881, *r* = 0.055) or pinprick score (*p* = 0.676, *r* = 0.152). tNAA/mI levels were similar in both patient groups (*p* = 0.396) and were not associated with pinprick score in patients with NP (*p* = 0.405, *r* = 0.242) and pain-free patients (*p* = 0.117, *r* = 0.527).

**Conclusions:**

Neuroinflammatory metabolite levels (i.e., tCho/mI) were elevated in patients with NP, its magnitude being associated with less cord atrophy and greater pain sensation (e.g., pinprick score). This suggests that patients with NP have more residual spinal tissue and greater metabolite turnover than pain-free patients. Neurodegenerative metabolite levels (i.e., tNAA/mI) were associated with greater cord atrophy but unrelated to NP. Identifying the metabolic NP signature provides new NP treatment targets and could improve patient stratification in interventional trials.

**Classification of evidence:**

This study provides Class II evidence that levels of magnetic resonance spectroscopy–identified metabolites of neuroinflammation were elevated in patients with SCI with NP compared to those without NP.

Spinal cord injury (SCI) is a life-changing event that generally leads to sensorimotor dysfunction below the injury level.^1^ Neuropathic pain (NP) arises as a secondary complication in >50% of the SCI population^2^ and has a negative impact on life quality.^3^ NP generally emerges within several months after SCI^4^ and is paralleled by trauma-induced neuroinflammation and neurodegeneration eventually affecting the entire neuraxis.^5–7^ However, NP-related metabolite level changes underlying neuroinflammatory and neurodegenerative changes are understudied, especially in the injured spinal cord.

Magnetic resonance (MR) spectroscopy (MRS) can noninvasively quantify metabolite levels,^8,9^ reflecting a biochemical profile of neuroinflammatory^10^ (i.e., elevated choline-containing compounds [tCho], cell membrane and myelin turnover marker,^11^ and myo-inositol [mI], glial cell marker^12,13^) and neurodegenerative^14^ (i.e., decreases in total N-acetylaspartate [tNAA], neuronal cell integrity marker^14^) processes. In patients with SCI with NP, higher tCho^15,16^ and mI^15–18^ were identified in key brain areas of pain processing compared to pain-free patients. In contrast, tNAA was decreased to a similar extent and unrelated to NP.^15^ In the injured cervical cord, Wyss et al.^9^ observed trauma-induced reductions in tNAA and tCho and elevated mI. Crucially, these neurodegeneration- and neuroinflammation-related changes showed clinicopathologic relationships.

However, how metabolic changes associated with neuroinflammation and neurodegeneration in the cervical cord relate to the presence of NP in patients with SCI is understudied. This study therefore aimed to investigate by means of spinal MRS metabolic changes at the cervical level C2/3 and their clinicopathologic associations in patients with SCI with chronic NP and pain-free patients with SCI.

## Methods

### Standard protocol approvals, registrations, and patient consents

The local ethics committee of Zurich approved the study protocol (KEK-ZH-No. 2014-610, PB_2016-00126, PB_2018-00937), which was conducted in accordance with the Declaration of Helsinki. All participants of this study were informed before study enrolment about the aim and procedure and provided written informed consent. A subset (9 paraplegic patients, 9 tetraplegic patients, and 11 healthy controls) of the data of this study was previously reported to assess metabolite ratios in the cervical spinal cord after SCI.^9^

### Primary research question

The primary research question of this study was whether levels of metabolites of neuroinflammation assessed by cervical cord MRS are elevated in patients with SCI with NP compared to those without NP.

### Classification of evidence

This article describes a diagnostic accuracy study that provides Class II evidence that levels of MRS-identified metabolites of neuroinflammation were elevated in patients with SCI with NP compared to those without NP.

### Participants

Twenty-four patients with SCI and 21 healthy controls were recruited between March 2016 and September 2018. Patients with SCI fulfilled the following inclusion criteria: (1) chronic traumatic injury (>1 year after injury), (2) no other neurologic or mental disorders, and (3) MRI compatible. Exclusion criteria of the study participants, including healthy controls, were preexisting neurologic, mental, or medical disorders affecting the outcome. One patient had to be excluded due to scan artifacts and another one because he did not fit in the neurovascular coil.

### Experimental design

#### MRI protocol

All study participants underwent MR measurements on a 3T Philips scanner (Achieva, release 3.2.3, Philips Healthcare, Best, the Netherlands) using a 16-channel SENSE neurovascular coil (Philips Healthcare). Spectra were acquired from the cervical spinal cord at level C2/3 (i.e., above the level of injury for all but 1 patient). The participants lay in the scanner in a head-first supine position, and the total scan duration was ≈45 minutes. MR measurement sequences included a survey scan, anatomic T1-weighted and T2-weighted (T2w) scans, and spectroscopic measurements.

At spinal level C2/3, T2w images (repetition time 3,000 milliseconds, echo time 120 milliseconds, flip angle 90°, in-plane resolution 0.5 × 0.5 mm, slice thickness 3.2 mm) were used to place the spectroscopic voxel (dimensions: 6 × 9 × 35 mm, 1.9 mL). The metabolite cycling (MC) technique^19^ was then applied in combination with inner volume saturated PRESS^20^ using broadband outer volume suppression pulses with optimal flip angles^21–23^ as reported previously.^9^ In addition, we used a second-order projection-based shimming routine.^24,25^ To reduce patient motion to a minimum, the spectroscopic acquisition was split into measurement acquisition blocks of 128 or 256 signal averages, and voxels were readjusted on the basis of an updated T2w image (repetition time 2,000–2,500 milliseconds [heartbeat triggered], echo time 30 milliseconds, number of total signal averages 512, spectral bandwidth 2,000 Hz, readout duration 512 milliseconds).

#### MRI postprocessing

##### Quality of MRS measurements

**Figure 1:**
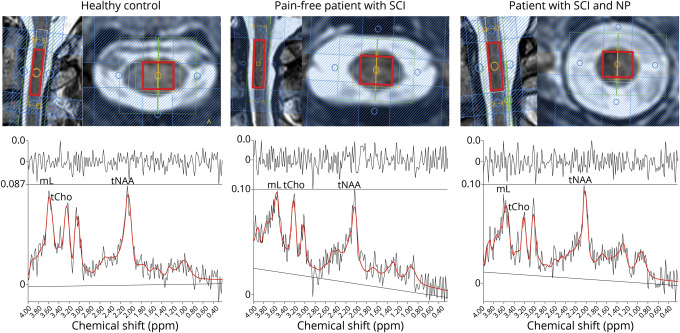
Representative planning images of spectroscopic voxel placement and metabolite spectra Magnetic resonance spectroscopic voxel of interest and representative spectra including the fitting (red lines) and original signal (gray lines) are shown for a healthy control, a pain-free patient with spinal cord injury (SCI), and a patient with SCI with neuropathic pain (NP). mI = myo-inositol; tCho = total choline-containing compounds; tNAA = total N-acetylaspartate.

**Figure 2:**
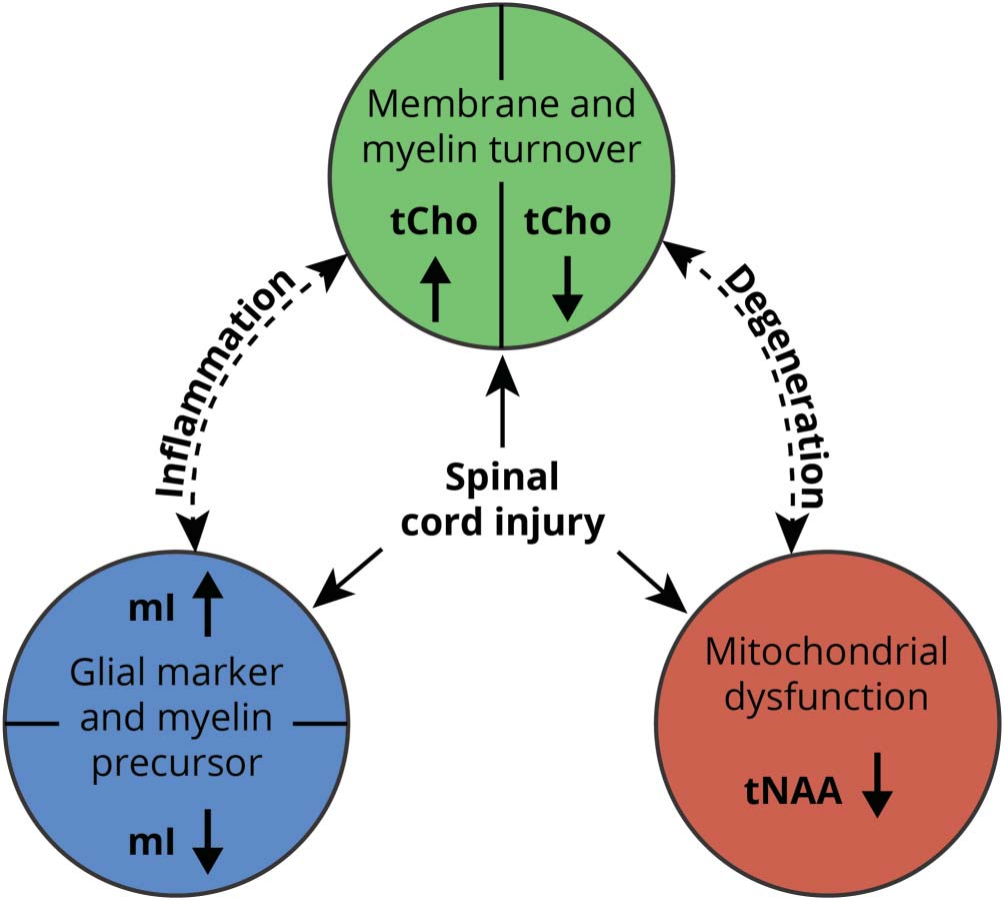
Overview of metabolites involved in SCI-induced processes Illustrative oversimplified scheme showing the ongoing principal structural and cellular changes after spinal cord injury (SCI) and their corresponding representative metabolites. Elevated total choline-containing compounds (tCho): Stanwell et al.,^16^ Chang et al.,^12^ and Widerström-Noga et al.^15^; lower tCho: Wyss et al.^9^ Elevated myo-inositol (mI): Pattany et al.,^18^ Chang et al.,^12^ Widerström-Noga et al.,^15^ and Wyss et al.^9^; lower mI: Fisher et al.^40^ Lower total N-acetylaspartate (tNAA): Wyss et al.^9^

The spectroscopic measurement blocks were checked for motion artifacts. No measurement block had to be excluded. Metabolic concentration values could be determined for all metabolites (tCho, tNAA, and mI) in all measurements. Representative planning images and spectra are shown for a healthy control, a pain-free patient with SCI, and a patient with SCI with NP (figure 1). An oversimplified overview of the molecules measured and the presumed interactions is illustrated in figure 2.

##### Postprocessing and quantification of spectroscopic data

We used MATLAB 2014b (MathWorks, Inc, Natick, MA) and the commercially available MRecon framework (version 3.0.530, GyroTools LLC, Zurich, Switzerland) for reconstruction of the spinal cord spectroscopic data from the raw data of the scanner. Eddy current, phase, and frequency alignment parameters were extracted from the unsuppressed water spectrum reconstructed from the MC series. Subsequently but before merging of all acquisition blocks, eddy current correction and frequency alignment were applied to the metabolite spectra reconstructed from the MC subseries by an add-subtract scheme as previously reported.^19^ Before quantification, truncation and zero filling were used in the time domain after 200 milliseconds. Relative values of metabolite concentrations were obtained by scaling them to mI (as previously reported^9^) because absolute quantification by internal spinal water referencing is not reliably feasible due to the pulsating surrounding CSF as previously shown in supplementary figure 1 in reference 9. Spectroscopic data of the cervical spinal cord were fitted and quantified with LCModel.^26^ A basis set for echo time of 30 milliseconds was used, including simulated basis set model data of N-acetylaspartate, N-acetyl-aspartyl-glutamate, glutamate, glutamine, glycerophosphocholine, phosphocholine, creatine, scyllo-inositol, and mI. Strongly overlapping resonance lines required a combination of the spectra of the following metabolites: N-acetylaspartate + N-acetyl-aspartyl-glutamate = tNAA, glycerophosphocholine + phosphocholine = tCho, and glutamate + glutamine is the combined expansion of Glx. A spectral range of 0.4 to 4.0 ppm was used in the fitting settings. To prevent artificial cutoff effects introduced by a cutoff value of 20%,^27^ we included metabolic ratios with Cramer-Rao lower bounds <100%.

#### Cross-sectional spinal cord area at cervical level C2/3

We used Jim 7.0 (Xinapse Systems, Aldwincle, UK) to assess the cross-sectional spinal cord area (SCA) at cervical level C2/3 on T2w images in all healthy control participants and patients with SCI. SCA was measured in 3 consecutive slices at the bottom of vertebra C2/3. In a first step, we determined a region of interest in the middle of the spinal cord on axial slices. In a next step, we used an active-surface model^28^ for the automatic calculation of SCA. Last, we manually adjusted the SCA outline in those slices of study participants in which automatic cord determination did not work properly (5 cases). Cross-sectional SCA could not be calculated for 1 patient due to motion artifacts during the MR acquisition.

### Clinical assessments

All patients with SCI were clinically assessed with a comprehensive clinical protocol including the International Standards for Neurologic Classification of Spinal Cord Injury (ISNCSCI) protocol for pinprick and light-touch scores^29^ and the European Multicenter Study About Spinal Cord Injury (EMSCI) pain questionnaire (version 4.2, emsci.org/).

With the ISNCSCI protocol, patients were classified as American Spinal Injury Association Impairment Scale (AIS) A (i.e., complete injury; no sensory or motor functions preserved in sacral segments), B, C, or D (i.e., incomplete injury), or AIS E (i.e., no functional impairment) according to the neurologic classification of SCI. The neurologic level of injury (NLI) is defined as the uppermost segment with neurologically intact motor and sensory scores.

In all patients with SCI, the EMSCI pain questionnaire was used as a screening tool for pain after SCI. This pain questionnaire examines various aspects of pain (e.g., current pain intensity, average and maximal pain intensity during the last week, location and quality of pain, intensity of allodynia and paresthesia). The pain intensity was rated with an 11-point numeric rating scale with 0 indicating no pain and 10 indicating the worst imaginable pain. In addition, the type of pain was explored and grouped into nociceptive (i.e., musculoskeletal or visceral) or NP (i.e., at, below, or at and below the lesion level). To be classified as at-level NP, ongoing pain had to be experienced within the 3 dermatomes below the NLI, 1 dermatome above the NLI, or both. Below-level NP, on the other hand, was defined as NP >3 dermatomes below the NLI.^30^ All patients completed the full protocol.

### Statistical analysis

Statistical analyses were performed with the R software package^31^ (version 3.4.3) and Stata software (version 14.2; StataCorp LP, College Station, TX), which was also used for visualization. We applied a 1-way analysis of variance followed by a Bonferroni post hoc test for pairwise comparison of healthy controls, patients with SCI with NP, and pain-free patients with SCI regarding their age at the time point of measurement. Unpaired 2-tailed *t* tests were used to compare the pinprick score and time since injury between patients with NP and pain-free patients. Between-group comparison graphics of metabolic ratios show box plots including the median and quartiles. These group differences between the healthy control group, patients with SCI with NP, and pain-free patients were assessed for metabolite concentration ratios of tCho/mI and tNAA/mI at spinal level C2/3 with a Kruskal-Wallis test followed by pairwise Mann-Whitney *U* tests. We used Spearman rank correlation tests to investigate associations between the amount of cervical cord atrophy in patients with SCI, assessed by the cross-sectional SCA at cervical level C2/3, and metabolite concentration changes at the same level. Last, we analyzed relationships between clinical measures of pain perception (pinprick score) and the metabolite ratio of tCho/mI at C2/3 using Spearman rank correlation tests.

The confidence interval (CI) was set to 95%. Results with an uncorrected value of *p* ≤ 0.05 were regarded as significant. We did not adjust for multiple testing to reduce type II errors, which potentially increased type I errors. Age was included as a covariate of no interest in the statistical model to adjust for age dependency.

### Data availability

Anonymized grouped data will be shared by request from a qualified investigator.

## Results

### Demographics and clinical characteristics

Fourteen patients with SCI with NP (12 men, age [mean ± SD] 52.2 ± 10.5 years, years since injury 11.3 ± 9.2), 10 pain-free patients with SCI (10 men, age 50.0 ± 10.3 y, years since injury 18.4 ± 10.5), and 21 healthy control participants (18 men, age 46.0 ± 11.2 y) were recruited. From all patients with SCI, 12 were classified as functionally complete (AIS A) and 12 as functionally incomplete (AIS grades B–D) (table). The NP group consisted of 8 tetraplegic and 6 paraplegic patients, while the pain-free group consisted of 5 tetraplegic and 5 paraplegic patients. Pinprick score was 64.3 ± 31.9 and 46.9 ± 20.6 for patients with SCI with NP and pain-free patients, respectively (*p* = 0.145, 95% CI −41.3 to 6.5). Patients with NP reported a mean pain intensity of 4.3 (SD 1.7, minimum 1, maximum 7) on a scale from 0 to 10. Mean age at injury was 40.9 years (SD 14.0 years) for the patients with SCI with NP and 31.6 years (SD 13.5 years) for the pain-free patients with SCI. The participants' mean age did not differ between any of the groups (*p* = 0.245). There was no significant difference in mean time since injury in years between the patient groups (*p* = 0.092, 95% CI −1.3 to 15.5).

**Table:**
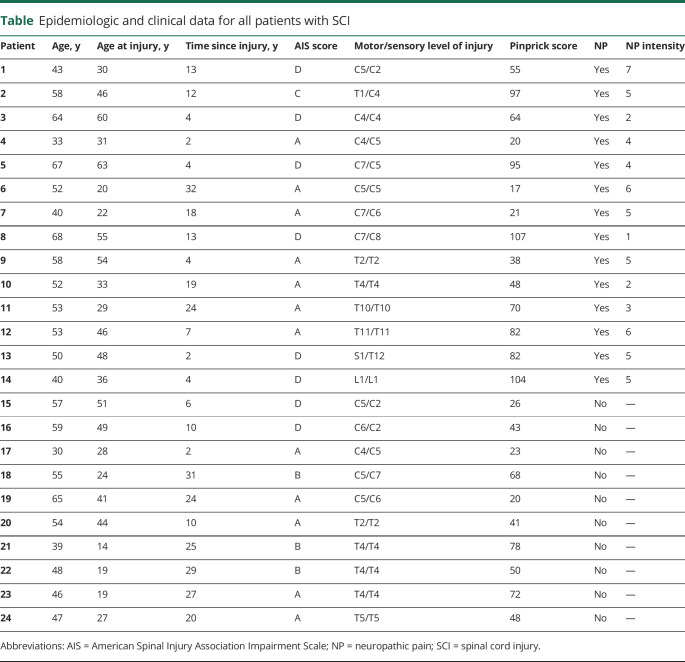
Epidemiologic and clinical data for all patients with SCI

### Changes of choline-containing compounds to mI

**Figure 3:**
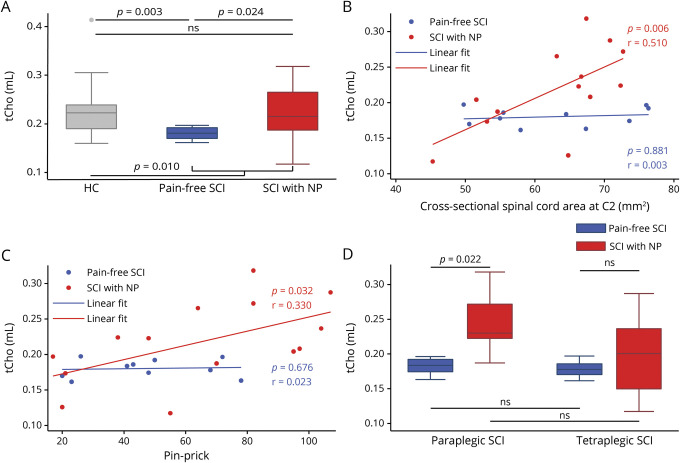
Group comparison of tCho/mI levels and correlation with spinal cord atrophy and clinical outcome measure (A and D) Group differences of the ratio of total choline-containing compounds to myo-inositol (tCho/mI) are shown for (A) healthy controls (HC; gray), pain-free patients with spinal cord injury (SCI; blue), and patients with SCI with neuropathic pain (NP; red) and for (D) paraplegic and tetraplegic patients with SCI in the pain-free (indicated in blue) and NP (indicated in red) groups separately. (B and C) Rank correlation graphs showing the associations of tCho/mI levels with cross-sectional spinal cord area at spinal level C2/3 (B) and pinprick score (C) for all patients with SCI. Pain-free patients with SCI are indicated by blue dots, patients with SCI with NP by red dots. Uncorrected *p* values are reported for significant differences. ns = not significant; tCho = total choline-containing compounds.

We first confirmed that there was a difference in tCho/mI levels between groups (*p* = 0.010) as previously reported for patients with SCI and healthy controls in a subset of this population.^9^ Patients with SCI with NP had a tCho/mI ratio (*p* = 0.699, 95% CI −0.049 to 0.029, figure 3A) similar to that of healthy controls but an elevated tCho/mI ratio (*p* = 0.024, 95% CI 0.007–0.075) compared to pain-free patients. In turn, the tCho/mI ratio was lower in pain-free patients with SCI compared to healthy controls (*p* = 0.003, 95% CI −0.069 to −0.016).

In patients with SCI with NP, a higher tCho/mI ratio was positively associated with a larger cross-sectional SCA (*p* = 0.006, *r* = 0.714, 95% CI 0.403–1.0236, n = 13, figure 3B) and a higher pinprick score (*p* = 0.032, *r* = 0.574, 95% CI 0.222–0.927, n = 14, figure 3C) but not with NP intensity (*p* = 0.406, *r* = −0.241, 95% CI −0.819 to 0.336, n = 14). In pain-free patients with SCI, the tCho/mI ratio was not related to cross-sectional SCA (*p* = 0.881, *r* = 0.055, 95% CI −0.709 to 0.818, n = 10) or pinprick score (*p* = 0.676, *r* = 0.152, 95% CI −0.637 to 0.940, n = 10).

To assess the effect of lesion level, we found that the tCho/mI ratio was not different between paraplegic and tetraplegic patients with NP (*p* = 0.138, 95% CI −0.021 to 0.111, figure 3D) and paraplegic and tetraplegic pain-free patients (*p* = 0.835, 95% CI −0.023 to 0.026). Within paraplegic patients, the tCho/mI ratio was elevated in patients with NP compared to pain-free paraplegics (*p* = 0.022, 95% CI 0.013–0.126), but this effect was not seen within the tetraplegic patient group (*p* = 0.421, 95% CI −0.052 to 0.090).

### Changes of tNAA to mI

**Figure 4:**
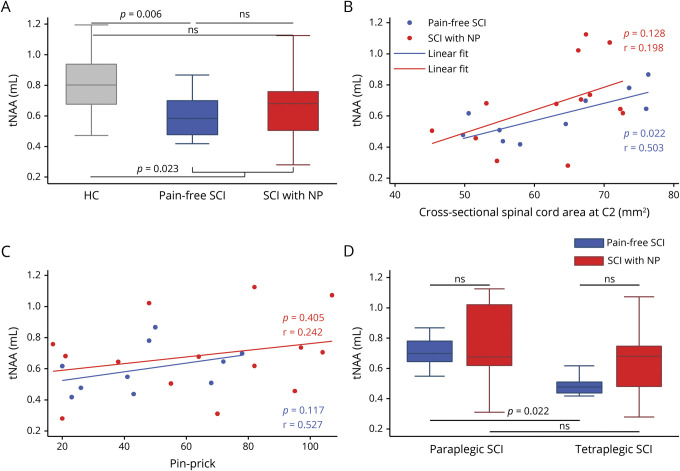
Group comparison of tNAA/mI levels and correlation with spinal cord atrophy and clinical outcome measure (A and D) Group differences of the ratio of total N-acetylaspartate to myo-inositol (tNAA/mI) are shown for (A) healthy controls (HC; gray), pain-free patients with spinal cord injury (SCI; blue), and patients with SCI with neuropathic pain (NP; red) and for (D) paraplegic and tetraplegic patients with SCI in the pain-free (indicated in blue) and NP (indicated in red) groups separately. (B and C) Rank correlation graph showing the association of tNAA/mI levels with cross-sectional spinal cord area at spinal level C2/3 (B) and pinprick score (C) for all patients with SCI. Pain-free patients with SCI are indicated by blue dots; patients with SCI with NP, by red dots. Uncorrected *p* values are reported for significant differences. ns = not significant.

We first confirmed that there was a difference in tNAA/mI levels between groups (*p* = 0.023) as previously reported for patients with SCI and healthy controls in a subset of this population.^9^ Patients with SCI with NP had a tNAA/mI ratio (*p* = 0.396, 95% CI −0.112 to 0.258, figure 4A) similar to that of pain-free patients, which did also not differ from that of healthy controls (*p* = 0.126, 95% CI −0.296 to 0.045). In pain-free patients, tNAA/mI levels (*p* = 0.006, 95% CI −0.378 to −0.071) were lower compared to healthy controls.

Lower levels of tNAA/mI were associated with a greater decrease of cross-sectional SCA in pain-free patients with SCI (*p* = 0.022, *r* = 0.709, 95% CI 0.252–1.166, n = 10, figure 4B), while no association was evident in patients with NP (*p* = 0.128, *r* = 0.445, 95% CI −0.028 to 0.918, n = 13). Pinprick score and tNAA/mI ratio were not associated in patients with NP (*p* = 0.405, *r* = 0.242, 95% CI −0.325 to 0.809, n = 14, figure 4C) or in pain-free patients (*p* = 0.117, *r* = 0.527, 95% CI 0.053–1.002, n = 10). There was no association between tNAA/mI levels and NP intensity in patients with NP (*p* = 0.783, *r* = −0.081, 95% CI −0.704 to 0.541, n = 14).

There was no difference in the tNAA/mI ratio between paraplegics with NP and tetraplegics with NP (*p* = 0.747, 95% CI −0.195 to 0.426, figure 4D). In pain-free paraplegics, the tNAA/mI ratio was higher compared to pain-free tetraplegics (*p* = 0.022, 95% CI 0.038–0.390). The tNAA/mI ratio was not different between paraplegic patients with NP and pain-free patients (*p* = 1.000, 95% CI −0.335 to 0.426), nor was it different between tetraplegic patients with NP and pain-free patients (*p* = 0.164, 95% CI −0.112 to 0.321).

## Discussion

This study shows metabolites of neuroinflammation and neurodegeneration and how their levels relate to NP in the spinal cord at cervical level C2/3 by means of noninvasive MRS. In particular, the ratio of tCho (i.e., a marker for an intact myelin and cell membrane turnover) over mI (i.e., a marker of activated glial cells) was increased in patients with SCI with chronic NP, but not in pain-free patients. This was distinct from markers of neurodegeneration (i.e., tNAA) that related to cord atrophy but not to NP. This study identified tCho/mI levels as a potential sensitive metabolite biomarker of NP in the injured cervical cord.

Regardless of NP, lower ratios of tNAA/mI and tCho/mI were evident in the atrophied cervical spinal cord^32^ of patients with SCI compared to healthy controls.^9^ This likely reflects ongoing neurodegeneration and activation of glial cells in the injured spinal cord,^33^ especially because the reduction of the metabolic ratios was greater in tetraplegic compared to paraplegic patients.

Aberrant activity of spinothalamic neurons in the spinal dorsal horn^34^ is believed to be a key player in the origin of NP,^35,36^ which manifests as an abnormal sensation of tingling and pricking (i.e., paresthesia), painful sensations induced by nonnoxious stimuli (i.e., allodynia), or increased or decreased responses to painful stimuli (i.e., hyperalgesia or hypoalgesia).^4^ In the spinal cord of patients with SCI with NP, this study shows higher tCho/mI ratios than in pain-free patients. In patients with SCI with NP, the concentration of tCho/mI was in the range of healthy controls. Higher tCho/mI ratios might relate to enhanced pain transmission in the spinal cord possibly due to an interplay of hyperactive spinothalamic neurons^35,37^ and inflammatory-induced glial activation.^37^ At the cellular level, this would relate to an increased membrane and myelin turnover (e.g., higher tCho)^11^ of spinothalamic neurons or activated glial cells (e.g., higher tCho and higher mI).^11,38,39^ Moreover, mI might be decreased and tCho/mI levels therefore increased due to less myelin breakdown in patients with SCI with NP.^40^ However, we are unable to disentangle the exact processes at the molecular level because both tCho and mI could drive the ratio differences. Nevertheless, we are confident that elevated tCho is significantly contributing to the ratio differences because it was shown that tCho was specifically elevated in distinct brain regions of the pain network (i.e., thalamus and anterior cingulate cortex) of patients with SCI with NP.^15,17^ These regions, together with the prefrontal cortex, sensorimotor cortex, and spinal cord, showed NP-associated bidirectional volume changes.^41,42^

To address the question of whether the metabolite level changes relate to NP or lesion level, we performed a subgroup analysis between paraplegic and tetraplegic patients. No lesion level–dependent difference in tCho/mI levels was evident. However, tNAA/mI levels were lower in tetraplegic compared to paraplegic pain-free patients, which is in agreement with our previous report,^9^ indicating that the magnitude of neurodegenerative processes is lesion level dependent. This finding is in line with a preclinical study reporting higher tNAA levels in a rabbit model of SCI with a light compared to a severe injury.^43^

As recently reported in a subset of this population,^9^ patients with SCI showed clinicopathologic relationships. However, previous analysis was performed regardless of NP. In this study, patients with SCI with NP showed a 3-way relationship between elevated tCho/mI ratios, less cord atrophy, and more preserved pain sensation (e.g., better pinprick score). This suggests that patients with SCI with NP have less degenerated spinal pathways (i.e., less cord atrophy and higher pinprick scores) and a greater metabolite turnover due to enhanced glial activation and proliferation.^38,39^ It is thus well imaginable that patients with SCI have a secondary complication (i.e., NP) compared to pain-free patients with SCI and show a higher potential for greater functional recovery due to the spared but somewhat disturbed tract and circuit function that is reflected by higher cervical cord tCho/mI levels. Similar to our results, an association between better recovery of pinprick score at 12 months and less cord atrophy immediately after the injury was previously reported in patients with SCI.^44^ In contrast, in pain-free patients with SCI, tCho/mI levels were not associated with cord atrophy or pinprick sensation. However, a lower tNAA/mI ratio was associated with greater cord atrophy, indicating processes of neurodegeneration. The 3-way relationship between metabolite levels, cord atrophy, and function speaks to the potential of levels of tCho/mI as a metabolite marker of inflammatory-induced glial activation and aberrant activity of spinothalamic neurons in NP states.

This study has limitations. First, the metabolites measured are presented as ratios^9^ to other metabolites and not as absolute values, unlike MRS values in the brain.^8^ This is due to the current lack of a reliable water reference signal in the spinal cord owing to the pulsating surrounding CSF (see supplementary figure 1 in reference 9). At present, it is therefore not possible to draw conclusions on single metabolites and their absolute values in spinal MRS acquisitions. In addition, ratios do not allow inferences about the directionality of the metabolite level changes. Moreover, the physiologic roles of the metabolites are manifold and still not completely known and proven. This impedes a reliable discrimination of neuroinflammatory and neurodegenerative processes from other mechanisms. In the future, we plan to look at spectroscopic data of specific pain areas within the brain and to compare the absolute metabolite levels to the ratios reported in the cervical cord. This could help us to better understand and identify which CNS regions and underlying mechanisms contribute to the development of NP. Second, in spinal MRS, the spectroscopic voxel covers the entire cord and thus cannot measure individual tracts. We therefore obtained metabolite signals derived from the gray and white matter simultaneously. Future studies would benefit from smaller, ideally tract-specific voxels. Moreover, more detailed pain assessments, including body pain drawings^45^ with the extent, intensity, and quality of the perceived pain, would enable a better characterization between metabolite turnover and the presence of NP. Third, our sample size was rather small. Given the fact that we were using MRS markers, we were not able to assess the extent of measurement error for the outcome measures. However, this does not invalidate our presented group comparisons and significant findings: it is possible that, in the future, variability due to measurement error may be reduced, in which case the required sample sizes would be smaller, reflecting the reduced measurement noise. Finally, sex was not equally represented in our patient cohort. However, the male-to-female patient ratio in this study closely reflects the general SCI population with a ratio of 4:1.^46^ Furthermore, healthy controls were age and sex matched.

This study identifies levels of tCho/mI, a marker of neuroinflammatory processes, as a discriminator of patients with SCI with NP from pain-free patients with SCI. Using spinal MRS, spinal cord atrophy assessments, and a clinical measure of pain sensation, we identified clinicopathologic associations. Thus, tCho/mI levels are a promising metabolite biomarker of neuroinflammation in the context of NP after SCI. Cervical cord MRS holds potential to be used in clinical trials for patient stratification, therapy monitoring, and outcome prediction.

## Glossary

AIS: American Spinal Injury Association Impairment Scale
CI: confidence interval
EMSCI: European Multicenter Study About Spinal Cord Injury
ISNCSCI: International Standards for the Neurological Classification of SCI
MC: metabolite cycling
mI: myo-inositol
MR: magnetic resonance
MRS: MR spectroscopy
NLI: neurologic level of injury
NP: neuropathic pain
tCho: total choline-containing compounds
tNAA: total N-acetylaspartate
SCA: spinal cord area
SCI: spinal cord injury
T2w: T2-weighted
